# The impact of anticipatory guidance on early childhood caries: a quasi-experimental study

**DOI:** 10.1186/s12903-018-0589-0

**Published:** 2018-07-27

**Authors:** Azhani Ismail, Ishak A. Razak, Norintan Ab-Murat

**Affiliations:** 1 Department of Community Oral Health and Clinical Prevention, Faculty of Dentistry, University of Malaya, 50603 Kuala Lumpur, Malaysia; 2 Klang Dental Clinic, Jalan Tengku Kelana, 41000 Klang, Selangor Malaysia; 3 Faculty of Dentistry, MAHSA University, 42610 Bandar Saujana Putra, Selangor Malaysia

**Keywords:** Anticipatory guidance, Oral health education, Early childhood caries, Oral health literacy

## Abstract

**Background:**

This study evaluated the impact of anticipatory guidance on the caries incidence of 2–3-year-old preschool children and their 4–6-year-old siblings, as well as on their mothers’ oral health literacy, as compared to the conventional Ministry of Health (MOH) programme.

**Methods:**

This quasi-experimental study was conducted at two government dental clinics in Batu Pahat District, Malaysia. The samples comprised of 478 mother-child-sibling trios (233 families in the intervention group, and 245 families in the control group). An oral health package named the Family Dental Wellness Programme (FDWP) was designed to provide dental examinations and oral health education through anticipatory guidance technique to the intervention group at six-month intervals over 3 years. The control group received the standard MOH oral health education activities. The impact of FDWP on net caries increment, caries prevented fraction, and mother’s oral health literacy was assessed after 3 years of intervention.

**Results:**

Children and siblings in the intervention group had a significantly lower net caries increment (0.24 ± SD0.8; 0.20 ± SD0.7) compared to the control group (0.75 ± SD1.2; 0.55 ± SD0.9). The caries prevented fraction for FDWP was 68% for the younger siblings and 63.6% for the older children. The 2–3-year-old children in the intervention group had a significantly lower incidence of white spot lesions than their counterpart (12% vs 25%, *p* < 0.05). At three-year follow-up, there were significant increments in the oral health literacy scores of mothers in the intervention group compared to the control group.

**Conclusion:**

The FDWP is more effective than the standard MOH programme in terms of children’s and siblings’ caries incidence and mother’s oral health literacy.

**Trial registration:**

ClinicalTrials.gov NCT03478748. Registered on March 26th 2018. Retrospectively registered.

## Background

Early Childhood Caries (ECC) is one of the most prevalent chronic childhood diseases [1, 2] and is characterized by the presence of one or more decayed (non-cavitated or cavitated), missing (because of caries), or filled tooth surfaces in any primary tooth in a child up to 6 years of age [3]. The effects of ECC extend beyond the child’s individual level as their health is also influenced by family and community factors [4]. Childhood often takes place at home, and parents, being the primary role models, have a major influence on their children’s oral hygiene and dietary practices. Indeed, studies have shown that oral health education (OHE) that targets parents, especially mothers, is beneficial in the prevention of ECC [5, 6]. One technique that has been suggested to increase the effectiveness of OHE is targeting the parents through anticipatory guidance. This proactive method, which is widely used in pediatric health care, is the process of providing practical, repeated rounds of developmentally appropriate health information about children to their parents, consequently maximizing parents’ roles in protecting their children from oral diseases [7, 8]. It avoids the use of paternalistic approach as in the traditional OHE, but instead involves interaction with parents in developing individualized OHE strategies. The American Academy of Pediatric Dentistry (AAPD) [3] recommends that parents with young children be provided with an anticipatory guidance programme that imparts information on the elimination of bottles in bed, early use of soft-bristled toothbrushes (with parental supervision), and limitation of a high-carbohydrate food intake after teeth have been brushed, with the first round scheduled within 6 months of the eruption of the first primary tooth [7, 8]. The use of this technique on first-time mothers, with information about the oral health of their infants, has been found to decrease the incidence of ECC [8].

The Ministry of Health (MOH), which is the main provider of oral health care in Malaysia, initiated the Preschool Oral Health Programme in 1984 with the aim of preventing ECC and maintaining a caries-free deciduous dentition among young children [9]. However, after more than three decades of implementing the programme, dental caries continues to be a major oral health problem among preschool children in Malaysia [10, 11]. One reason could be that the OHE given is mostly based on standard generic advice, and parental involvement is mostly ignored. This is despite the fact that interventions targeting parental empowerment towards their child’s oral health have been found to be beneficial in the prevention of ECC [12]. Another possible reason is that the intervention starts very late when some of these children are already experiencing high level of caries. Children age between 1 and 3 years old is most susceptible to caries [13]; it is therefore critical that early identification of risk factors and early dental interventions are carried out to these young children to help change their trajectory of oral health [14]. A new initiative is required to achieve the MOH target of having 50% caries-free children by the year 2020 and hence the Family Dental Wellness Programme (FDWP) was initiated. This oral health package that targets both mothers and their young children, comprises of tailor-made OHE messages delivered using anticipatory guidance technique at dental clinics at six-month intervals during a three-year period. This technique which adopts a wellness and self-care concept has been reported to be effective in the developed nations, but has yet to be tested in Malaysia which has a different sociocultural setting.

The aim of this study was to evaluate the effectiveness of the FDWP in reducing the incidence of ECC in children aged 2–3 years old, and their 4–6-year-old siblings who had high risk for caries, as well as the impact to their mothers’ oral health literacy. The outcome measures were net caries increment, caries prevented fraction, the incidence of white spot lesions, and mothers’ oral health literacy.

## Methods

### Participants

The sample size of this quasi-experimental study design was estimated based on the prediction that the FDWP would be able to reduce caries prevalence in children below 6 years old by at least 27% after 3 years’ implementation [15]. The only data available for caries prevalent in Batu Pahat district was on 6-year-olds children, which was 65.6% in 2012. In order to detect such a reduction at a significance level of 5% and a statistical power of 80%, and accounting for a 20% attrition rate, the minimum sample size required for the test group was 136. To overcome the cluster effect or sampling errors in selecting the control, a design effect by a factor of 1.8 was inflated to the control group. The design effect of 1.8 was assumed because of the variance in location of residences [16]. Hence, the minimum sample size required for the control group was 245.

The inclusion criteria for this study which comprised of mother-child-sibling trios were (i) children between the age of 2–3 years old, (ii) older sibling aged 4 to 6 years categorized as having high caries risk based on a Caries Risk Assessment tool adapted from the AAPD [17], and (iii) mothers who were willing to participate in the study. The older siblings were used as a proxy to depict caries trajectory of the younger children. If all risk and protective factors of caries remain the same or in other words, if no effective dental intervention was provided, the younger children will most likely develop caries experience as their older siblings when they reach preschool age. Children with medical conditions requiring special consideration as regards to their dental management and families with a high-mobility tendency were excluded from this study.

The samples for the intervention group were recruited from Batu Pahat Dental Clinic, where the FDWP was first initiated. However, the register of 2–3 year old receiving care at this clinic was very low, hence the register for preschool children (4–6 year-old) was used. A total of 1805 preschool children received dental care from this clinic. Of these, 245 children fulfilled the inclusion criteria and all were included as samples in the intervention group. The control group was recruited from preschool children under the care of a nearby dental clinic, and possible samples were matched to the 245 samples in the intervention group in terms of socio-demographic background (age, ethnicity, and family income) and other potential confounders such as family size and function. Four hundred preschool-aged children and their siblings were screened and the 245 children who fulfilled the inclusion criteria were included in the control group.

### Intervention

The FDWP is an OHE package that covers the following aspects: (i) cognitive component (for example, the provision of basic oral health knowledge using an anticipatory guidance technique); (ii) psychomotor component (for example, the demonstration of basic oral health skills such as toothbrushing skills with a hands-on practical); and (iii) attitude component (activities that help to improve the children’s attitude towards oral health). Children (along with their older siblings and mothers) who participated in the FDWP were required to make six-monthly visits to the dental clinic in the three-year study period.

We created Family Dental Wellness Zones in the clinic, which consist of six different zones with different OHE activities (Table 1). This was where children and their families were given a one-hour OHE session at each visit, by either the dentists or dental therapists. At baseline, subjects and their older siblings underwent oral examination and caries risk assessment procedures, and mothers were requested to complete the oral health literacy questionnaire. They then took part in activities conducted in the Family Wellness Zones. The activities conducted at each six-monthly visit were dental examination and OHE through anticipatory guidance technique. At three-year follow-up, the final dental examination was performed again on both the children and their siblings, and the mothers completed the same questionnaire on oral health literacy again.

**Table 1:** Family Dental Wellness Zones and their respective oral health education activities

Zone	Activities
Zone 1	Drawing of own teeth to assess the children’s knowledge of their own teeth and their associated tissues. Mothers and children were advised on the normal appearance of the child’s teeth so they can identify any oral problems should they occur in the future. Mothers were also trained to diagnose white spot lesions and were advised to do home oral exams about once a month.
Zone 2	Healthy snack games to assess mothers’ and children’s food preferences and to advise them about the effect of sugar on oral health, the importance of reading food labels, and age-appropriate healthy meals.
Zone 3	A wide range of toothbrushes and toothpastes were displayed and children were requested to choose, and state the reasons for their choices. Mothers and children were then advised on the appropriate usage of toothbrush and toothpaste, and the benefits of fluoride
Zone 4	Mothers were asked to apply plaque-disclosing solutions to their children’s teeth. A dental surgery assistant helped to explain about plaque to both mothers and children, and assisted mothers in calculating the plaque score.
Zone 5	Toothbrushing demonstrations and smiling contests amongst the samples and the younger siblings.
Zone 6	Multimedia interactive games on toothbrushing to highlight the importance of an effective brushing technique.

During the activities conducted in the aforementioned OHE zones, mothers were provided with practical, developmentally appropriate oral health information that would empower them to take the appropriate action to promote and maintain their children’s oral health. The anticipatory guidance process was guided by some trigger questions and based on the answers given by the mothers, relevant advices were provided. Repeated rounds of anticipatory guidance were given at the following six-monthly visits, using different trigger questions to mothers, based on the activities that they participated in during the visits. Samples in the control group received the standard MOH programme that focuses mainly on OHE at child level.

### Study instruments

At each visit, the children were examined on a dental chair using a disposable plane mouth mirror and a Community Periodontal Index probe. The oral health examination protocol followed the World Health Organization (WHO) guidelines [18]. However, some modifications were made to the WHO protocol, whereby Nyvad et al.’s [19] caries diagnostic criteria were incorporated to assess the presence of active white spot lesions. Only the status of the deciduous teeth was recorded. The examiner (AI) and two dental therapists who were involved in clinical examinations were trained and calibrated on the dft index and white spot lesion with a gold standard, who is a dental public health specialist and a recognized benchmark examiner based at the Malaysian Ministry of Health. The overall kappa scores were 0.89, 0.85, and 0.83 respectively for the dentist and the dental therapists. During data collection, intra- and inter-examiner re-calibration was performed on 20 children aged 5 years on successive days, and the kappa score obtained was above 0.8.

Mothers’ oral health literacy was assessed using the Dental Health Literacy Assessment instrument [20]. This instrument has been cross-culturally adapted for used on Malaysian population and was reported to be valid and reliable with a Cronbach alpha of 0.67 [21]. The questionnaire consists of three sections; Section 1 has 12 items that assess oral health knowledge; Section 2 consists of five questions testing mothers’ ability to comprehend healthcare instructions; and Section 3  evaluates mothers’ skills and motivation to care for their children’s oral health (39 items). The oral health literacy scores were calculated by summing up all items in Sections 1, 2 and 3 that range from 0 to 12, 0–5, and 0–39 respectively. Thus, the total scores for oral health literacy range from 0 to 56, with the higher score indicating better oral health literacy.

### Statistical analysis

Descriptive statistical analyses were performed to compare the sociodemographic characteristics of participants in both the intervention and control groups using chi square test and independent t-test, where appropriate. The mean dft, dt and ft between child and sibling group were analysed using Mann Whitney U, as the data were not normally distributed based on Kolmogorov-Smirnov test (*p* < 0.05). Comparison of net caries increment and the incidence of white spot lesion between child and sibling groups were analysed using independent-t-test. The total mean scores for mothers’ oral health literacy and the three respective domains (knowledge; comprehension; skills and motivation) were calculated based on Ludke et al.’s [20] recommendations. The mean differences of the OHL scores between groups were analysed using independent t-test as the data was normally distributed.

During dental examination, if a deciduous tooth was found to be missing at any follow-up, the score recorded during their last examination was used to denote its current status for that respective tooth. Caries prevented fraction was calculated based on the difference of the mean dft increment between the intervention and control groups over the mean increment of caries in the control groups. The mean dft increment was calculated by deducting the mean caries scores at baseline from those obtained after 3 years within the groups.

## Results

A total of 233 families in the intervention group and 245 families in the control group participated in the study. The response rate was 95.1 and 100% respectively for the intervention and control group (for both children and mothers). The overall response rate was 97.6%. There were no significant differences between the intervention and control group in terms of socio-demographic variables and caries status at baseline, as the samples for both groups were matched for their individual and family profiles, as well as possible potential confounders. The proportion of boys and girls was almost equal for both children and their older siblings. The majority was of Malay ethnicity, and most of their mothers were secondary school graduates (Table 2).

**Table 2:** Socio-demographic distribution at baseline for intervention and control group (*N* = 478)

Variable	Overall	Intervention	Control	*p-value*
	*N* (%)	*n* (%)	*n* (%)	
Gender of child:				
Male	215(45.0)	103 (44.2)	112 (45.7)	0.740^a^
Female	263(55.0)	130 (55.8)	132 (53.9)	
Age of child:				
2 years	240 (50.2)	118 (50.6)	122 (49.8)	0.853^a^
3 years	238 (49.8)	115 (49.4)	123 (50.2)	
Mean age of child^*^	2.5 ± 0.5	2.5 ± 0.5	2.5 ± 0.5	0.853^b^
Ethnicity of child:				
Malay	432 (90.4)	206 (88.4)	226 (92.2)	0.156^a^
Chinese	46 (9.6)	27 (10.6)	19 (7.8)	
Gender of older sibling:				
Male	237 (49.6)	111 (47.6)	126 (51.4)	0.408^a^
Female	241 (50.4)	122 (52.4)	119 (48.6)	
Age of older sibling:				
4 years	117 (24.5)	53 (22.7)	64 (26.1)	0.506^a^
5 years	155 (32.4)	81 (34.8)	74 (30.2)	
6 years	206 (43.1)	99 (42.5)	107 (43.7)	
Mean age of older sibling*	5.2 ± 0.8	5.2 ± 0.7	5.2 ± 0.8	0.765^b^
Mother’s highest education:				
Never been to school	13 (2.7)	5 (2.2)	8 (3.3)	0.222^a^
Primary school	43 (9.0)	19 (8.2)	24 (9.8)	
Secondary school	295 (61.7)	138 (59.2)	157 (64.1)	
Diploma, degree and above	127 (26.6)	71 (30.5)	56 (22.8)	

^a^Chi-square test; ^b^Independent t-test; significance level *p* < 0.05 *Mean and standard deviation

The FDWP children and siblings had a significantly (*p* < 0.01) lower net caries increment (0.24, SD = 0.8; 0.20, SD = 0.7) than the control group (0.75, SD = 1.2; 0.55, SD = 0.9) at three-year follow-up (Table 3). At the end of the study period, a large proportion of the caries experience is accounted for by the filled component which is significantly higher among the 4–6 year old siblings in the FDWP group. The caries prevented fraction for FDWP was 68 and 63.6% for the younger and older siblings respectively. Children aged 2–3 years old who underwent the FDWP had significantly lower incidence rates of white spot lesion (12%) than their counterpart in the control group (25%) at three-year follow-up (*p* < 0.05). However, there was not much difference in the incidence rate of white spot lesion among the older siblings in the intervention and control groups (3% versus 4%), and the result was not statistically significant (Table 4).

**Table 3:** Net caries increment and caries prevented fraction of 2–3-year-old children and their older siblings in control and intervention group (*N* = 478)

	Intervention (*n* = 233)			Control (*n* = 245)			*p*-value^a^	Caries prevented fraction (%)
	Baseline	Follow-up	Net increment	Baseline	Follow-up	Net increment		
	Mean ± SD			Mean ± SD				
2–3-year-old								
dft	0.94 (1.6)	1.18 (1.8)	0.24(0.8)	1.15 (1.6)	1.90 (1.9)	0.75 (1.2)	< 0.001	68.0
dt	0.76 (1.2)	0.34 (0.8)	−0.42 (0.9)	0.91 (1.4)	0.87 (1.4)	0.04 (1.2)	< 0.001	
ft	0.18 (0.6)	0.84 (1.3)	0.66 (1.1)	0.24(0.8)	1.04 (1.2)	0.79 (1.3)	0.021	
4–6-year-old								
dft	2.45 (2.1)	2.60 (3.0)	0.20 (0.7)	2.08 (2.3)	2.63 (2.4)	0.55 (0.9)	< 0.001	63.6
dt	2.07 (2.6)	0.97 (1.7)	−1.17 (1.8)	1.57 (1.8)	1.29 (1.5)	− 0.28 (1.3)	< 0.001	
ft	0.33 (0.9)	1.70 (2.0)	1.37 (1.7)	0.51 (1.1)	1.34 (1.5)	0.83 (1.2)	< 0.001	

^a^Mann-Whitney U; significance level *p* < 0.05

**Table 4:** Incidence of white spot lesions of 2–3-year-old children and their older siblings in the control and intervention group (*N* = 478)

	Group	Number of new cases of white spot	Number of children at risk	Incidence of white spot lesions	Incidence rate (%)	*p*-value ^a^
2–3 year-old children	Intervention	24	196	0.12	12	< 0.001
	Control	53	211	0.25	25	
4–6 year-old children	Intervention	5	159	0.03	3	0.621
	Control	6	166	0.04	4	

^a^Independent T-test; significance level *p* < 0.05

Table 5 shows the effect of anticipatory guidance on mothers’ oral health literacy at baseline and follow-up between the two groups. There was baseline equivalence of mothers in the control and intervention group for all oral health literacy variables prior to intervention (*p* > 0.001). However, at the end of the intervention, there were significant increments of scores in the knowledge, comprehension, skills, and motivation sections, as well as the overall oral health literacy scores of mothers in the intervention group, as compared to the control group. The magnitude of change in the mean scores for the overall oral health literacy of mothers in the intervention group pre and post intervention was significantly higher (7.70 ± 0.4) than that of the control group (0.46 ± 0.5).

**Table 5:** Mean differences of mothers’ oral health literacy scores between the intervention and control groups

Variable	Intervention Mean (SD)	Control Mean (SD)	Mean differences between group, Mean (SD)	95% CI	*p*-value^a^
Knowledge:					
Baseline	6.73 (1.9)	6.47 (2.1)	0.25 (0.2)	−0.11-0.62	0.167
Post-intervention	9.07 (1.6)	6.80 (2.1)	2.26 (1.7)	1.93–2.59	< 0.001
Comprehension:					
Baseline	4.27 (1.0)	4.04 (1.1)	0.23 (0.01)	0.04–0.41	0.006
Post-intervention	4.71 (0.5)	4.17 (1.0)	0.57 (0.7)	0.43–0.70	< 0.001
Skills & motivation:					
Baseline	26.13 (4.5)	26.15 (5.2)	1.94 (2.1)	−0.90- 1.86	0.968
Post-intervention	32.92 (2.5)	28.09 (4.4)	4.87 (0.3)	4.23–5.5	< 0.001
Oral health literacy:					
Baseline	37.13 (5.7)	36.67 (6.5)	0.46 (0.5)	−0.64 – 1.57	0.411
Post-intervention	46.74 (3.4)	39.05 (5.7)	7.70 (0.4)	6.85–8.55	< 0.001

^a^Independent t test; significance level *p* < 0.05

## Discussion

This study assessed the effect of anticipatory guidance provided to mothers in the FDWP on their children’s caries increment and caries prevented fraction, and their own oral health literacy. The main findings showed that the intervention not only resulted in reduced caries increment among the younger children in the intervention group but the positive effect was also seen in the older siblings who were already diagnosed as having high caries risk. Children in the intervention group and their siblings also had a higher prevented fraction of 63.6 and 68.0% respectively than the control group at follow-up. The values of the prevented fraction in the present study are considered high compared to findings from the systematic reviews of OHE intervention aimed to reduce ECC [22], which ranged from 18 to 93%. These findings suggest that anticipatory guidance provided to mothers is beneficial in the prevention of ECC amongst their 2–3-year-old children, as well as the caries-prone older siblings in the family.

Mothers, being the primary role model in shaping children’s behavior, have major influences on their children’s oral habits and practices. Particularly, their education level is one of the important socioeconomic indicators that affect the incidence of ECC of their children. Highly educated mothers were reported to have higher positive attitudes and stronger intentions to control children’s sugar intake, as compared to low-educated parents [23]. Hallet and Rourke [24] also proved that the prevalence and severity of ECC is linked to decreasing level of mother’s education. The OHE activities provided in the Family Wellness Zones and the trigger questions and related advice given to mothers in this study have been shown to improve their knowledge, comprehension, skills, and motivation to care for their child’s oral health. The knowledge and skills that they acquired may translate into their own positive oral health behaviors, which have been shown to exert a significant positive influence on children’s toothbrushing habits and caries experience [25]. Although parental-related factors have been acknowledged to influence the development of ECC, studies that assessed the association of individual factors and social determinants seem to attract more attention in the ECC research areas [12]. Children’s greatest social support initially comes from their family; hence it is important to empower families, especially mothers, to take control of their children’s oral health.

Anticipatory guidance assists mothers or parents to anticipate any changes or potential oral health problems that may occur in relation to their children’s health and oral development. The AAPD [3] advocates that anticipatory guidance be given to expectant mothers and parents of infants and young children and age-appropriate advice is provided to prevent or control oral diseases. For young children aged between 2 and 6 years old, appropriate oral health advice should include counseling sessions on oral hygiene, dietary, injury-prevention, and non-nutritive habits. In our study, we focused only on oral hygiene and dietary advice, as this information is related to caries incidence, which is our main study objective.

Recently, there have been initiatives to replicate the FDWP in other government dental clinics in Malaysia. The recently initiated FDWP can be strengthened by including anticipatory guidance initiatives that are advocated by the AAPD, as mentioned above. However, the uptake of anticipatory guidance in dental clinics globally has been lukewarm as compared to that by their medical counterparts, and the impact of this method on children’s oral health has not been well studied [26]. Ramazani et al. [27] found that anticipatory guidance improved Iranian expectant mothers’ knowledge about maternal, infant, and toddler’s oral health. Plutzer and Spencer [8] evaluated the effect of anticipatory guidance provided to expectant mothers and found that the incidence of ECC among their children in the intervention group (1.7%) is significantly lower than in the control group (9.6%). It is not possible to compare the incidence rate of ECC among children in our study to that of the latter study, as they assessed ECC at the relatively early age of 20 months old, which could partly explain the lower incidence observed. In addition, interventions started early in that study with expectant mothers being provided with oral health information during pregnancy, which may assist them to set up proper oral health habits for their children earlier.

The strength of using anticipatory guidance is that it does away with the standard generic advice that most dentists usually offer in their clinic. Instead, this technique forces interaction between dentists and parents in obtaining the children’s oral health-related development information [7], which is then used to provide oral health anticipatory advice matched to each child’s characteristics. This customization of OHE has been shown to increase clients’ interest and positively affect their cognitive responses to the information received [28]. Cognitive responses stimulated by tailored health messages have been shown to significantly lead to subsequent behavioral intention and actual behavior change [29]. In our intervention, the anticipatory guidance was delivered by a verbal face-to-face technique, without any printed health education take-home messages. There is some evidence that anticipatory guidance provided by a face-to-face technique has a better outcome than information given using pamphlets [27]. Most new mothers now are from the millennial generations and are users of social technology; hence, it would be interesting to assess the potential of using social media in enhancing personalized oral health communication amongst these web-savvy generations.

Although the effectiveness of the FDWP in reducing caries incidence among young children and the improvement in their mothers’ oral health literacy has been proven in this study, its incorporation into the mainstream Ministry of Health programme will be strongly dependent on the political will of the Ministry. As it involved individualized strategies of intervention which is labour intensive, the cost of its application may deem to be prohibitive and may discourage its formal adoption. A possible trade off would be that this programme be restricted to high caries risk communities which would make it to be cost effective.

Several limitations restrict the interpretability of the present study. Firstly, the influence of social and mass media in the dissemination of information cannot be ignored. This may result in mothers in the control group also obtaining the oral health-related information received by mothers in the intervention group. Hence, if this influence is disregarded, the impact of the anticipatory guidance would have been more significant. Secondly, the control group was recruited from a dental clinic under the same administration of Batu Pahat Oral Health Division, which means that the clinics are in close proximity to one another. This was done to ensure that subjects in both intervention and control groups had similar socio-demographic characteristics. Hence, the occurrence of a halo effect or diffusion could exist, as mothers in the control group may have received the anticipatory guidance from mothers in the test group or they may also have visited the clinic involved in the interventions and obtained the information indirectly. Thirdly, the FDWP children and siblings had a significantly lower net caries increment than the control group at three-year follow-up with a large proportion of the caries experience being accounted for by the filled component. It cannot be discounted that both groups may be exposed to different criteria of restorative dental care. However this is unlikely as both groups come under the management of the same Senior Dental Officer in charge of the administrative district. The use of a quasi-experimental study design also limited the generalizability of the findings and reduced its internal validity. Equality of the two groups was a concern because random assignment (randomization) is absent. We tried to minimize participant differences by selecting participants as similar as possible in terms of socio-demographic and oral health status at baseline. Barring these limitations, the results of this pilot study show that anticipatory guidance provided to mothers assists in reducing the prevalence of caries of the children, and improved the mothers’ oral health literacy. To ensure the FDWP remains relevant and sustainable, both dentists and dental therapists must undergo continuous training to increase and update their knowledge, to improve their oral health promotion skills, and to maintain their motivation in ensuring the success of this programme.

## Conclusions

The anticipatory guidance technique used in the FDWP was effective in reducing caries incidence among young children aged six and below, and improved mothers’ oral health literacy after 3 years of implementation. It is recommended that this technique is incorporated in OHE activities targeting children.

## Abbreviations

AAPD: The American Academy of Pediatric Dentistry
ECC: Early childhood caries
FDWP: Family Dental Wellness Programme
MOH: Ministry of Health
OHE: Oral health education
WHO: World Health Organization
